# Impact of Changes in Detection Effort on Control of Visceral Leishmaniasis in the Indian Subcontinent

**DOI:** 10.1093/infdis/jiz644

**Published:** 2019-12-16

**Authors:** Luc E Coffeng, Epke A Le Rutte, Johanna Muñoz, Emily R Adams, Joaquin M Prada, Sake J de Vlas, Graham F Medley

**Affiliations:** 1 Department of Public Health, Erasmus MC, University Medical Center Rotterdam, Rotterdam, The Netherlands; 2 Department of Epidemiology and Public Health, Swiss Tropical and Public Health Institute, Basel, Switzerland; 3 University of Basel, Basel, Switzerland; 4 Department of Tropical Disease Biology, Liverpool School of Tropical Medicine, Liverpool, United Kingdom; 5 School of Veterinary Medicine, Faculty of Health and Medical Sciences, University of Surrey, Guildford, United Kingdom; 6 Centre for Mathematical Modelling of Infectious Disease and Department of Global Health and Development, London School of Hygiene and Tropical Medicine, London, United Kingdom

**Keywords:** visceral leishmaniasis, improved case detection, mortality, resurgence, transmission dynamics, mathematical modeling

## Abstract

**Background:**

Control of visceral leishmaniasis (VL) on the Indian subcontinent relies on prompt detection and treatment of symptomatic cases. Detection efforts influence the observed VL incidence and how well it reflects the underlying true incidence. As control targets are defined in terms of observed cases, there is an urgent need to understand how changes in detection delay and population coverage of improved detection affect VL control.

**Methods:**

Using a mathematical model for transmission and control of VL, we predict the impact of reduced detection delays and/or increased population coverage of the detection programs on observed and true VL incidence and mortality.

**Results:**

Improved case detection, either by higher coverage or reduced detection delay, causes an initial rise in observed VL incidence before a reduction. Relaxation of improved detection may lead to an apparent temporary (1 year) reduction in VL incidence, but comes with a high risk of resurging infection levels. Duration of symptoms in detected cases shows an unequivocal association with detection effort.

**Conclusions:**

VL incidence on its own is not a reliable indicator of the performance of case detection programs. Duration of symptoms in detected cases can be used as an additional marker of the performance of case detection programs.

Visceral leishmaniasis (VL), also known as kala-azar, is a neglected tropical disease caused by single-celled *Leishmania* parasites that are transmitted by sandflies [1]. On the Indian subcontinent, VL is considered entirely anthroponotic. Once infected, a small percentage of individuals develop symptoms that are fatal when left untreated. After successful treatment, 5%–20% of cases develop a skin condition known as post–kala-azar dermal leishmaniasis (PKDL), which lasts several years if left untreated [2]. Transmission is driven by cases of symptomatic infection and PKDL; asymptomatic cases most likely do not infect sandflies or to a much lower extent [3, 4].

The World Health Organization 2020 target for control of VL on the Indian subcontinent is defined as <1 detected VL case per 10 000 population per year at the (sub)district level (minimum 35 000 population; median size of 200 000) [5]. Control strategies rely on prompt detection and treatment of VL cases, and vector control in the form of indoor residual spraying of insecticide [5], although several studies question the impact of indoor residual spraying on VL incidence [6, 7]. Strategies to improve the promptness of detection include provision of diagnostics, raising clinical and community awareness, and more recently, active case detection given that cases tend to be clustered in time and space [8, 9]. Detection success is generally measured through the average time between onset of symptoms and specific diagnosis, and this has reduced substantially, although it still shows substantial variability [10]. Given this variability, it is surprising that, to our knowledge, no consideration has been given to the impact of population coverage of improved detection programs and/or reductions in detection delay on achievement of control. It should also be noted that it is only possible to measure the diagnostic promptness in detected cases.

Given the drop in the number of VL cases on the Indian subcontinent due to large-scale control efforts since 2010, achievement of the control target seems within reach in many regions [11–13]. However, if control efforts relax following the achievement of this target, the sustainability of VL control could be at stake [14]. Here, we hypothesize that in some situations, relaxation of detection efforts will lead to an apparent (temporary) achievement of the control target, whereas the true, underlying epidemiological situation is worsening. Such a relaxation could occur through lack of clinical awareness, reduction in resources due to political complacency, or diversion of resources from detection to another form of control.

Mathematical models of VL transmission are increasingly used for planning and assessing the efficacy of interventions and evaluating the intensity and timescale required to achieve set targets [13, 15]. In this study, we use a mathematical model for transmission and improved detection of VL to predict the impact of reduced detection delays and/or increased population coverage of the detection programs on VL incidence and mortality.

## METHODS

### Model Structure

In this study, we employed a simplified version of earlier transmission models [16–18], keeping only the processes in the model that are relevant to the impact of improved detection of VL cases. See Supplementary Appendix A for a schematic representation of the model structure. In the model, susceptible individuals who are infected with the *Leishmania* parasite first enter a stage of latent infection, which is asymptomatic and noninfectious. Three percent of latent infections progress to developing symptomatic VL, which is diagnosable and infectious, and the remainder recover without treatment [19]. The current definition of VL implies that individuals have clinical symptoms (fever) for 2 weeks prior to being diagnosable. Here, the detection and subsequent treatment of symptomatic cases were assumed to occur at a constant rate, so that the resulting distribution of detection delays reflects the high variation in reported treatment delays in India [20]. The competing risk of dying from untreated VL was assumed to increase with duration of symptoms, which was captured using the “linear chain trick” [21] to model progression until death as an Erlang distribution with shape 3. Together, the competing risks of being detected vs dying determine the proportion of VL cases that die undetected, the average time till death, and the duration of symptoms in the detected cases. A baseline situation with “standard” detection effort was defined as a situation in which half of the VL cases die undetected, and those who die have symptoms for an average duration of 150 days. These figures are completely unobserved but are consistent with reports of the case ascertainment [22, 23], and were uniquely reproduced by setting the baseline case detection rate to 365/243 (ie, an average detection delay of 243 days in absence of excess mortality) and the annual mortality rate due to untreated VL to 365/189 (ie, an average duration until death of 189 days in absence of any detection effort). These rates translate to an average detection delay of about 8 months in absence of VL-related mortality, an average duration of symptoms before death of about 6 months in absence of any detection effort or healthcare-seeking behavior, and an average detection delay in detected cases of 92 days.

To simulate the potential impact of an improved detection program, we stratify the population of symptomatic cases into 2 fractions: one covered by the improved detection program (ie, shorter treatment delay), and the other covered by the baseline detection rate. The 2 groups are subject to the same risk of dying from untreated VL. All detected VL cases are assumed to be successfully treated and reach the dormant stage, which lasts on average 21 months [24–26], after which most will recover completely. Five percent of individuals in the dormant stage will develop PKDL [2], which lasts 5 years on average [24]. Individuals who recover fully from the dormant stage or PKDL are assigned to the fully recovered state, which we assume cannot be infected and lasts 5 years on average [18], after which they become susceptible again. Only VL cases and PKDL cases are considered to be infectious and contribute to transmission [3]. The background mortality rate due to other causes was based on the average expected lifespan at birth in rural Bihar, as reported for 2010–2014 by the Indian Census Office [27]. We did not consider age and population growth in our model, as these were not deemed relevant for the diagnostic process or VL transmission dynamics when predicting short-term trends.

The transmission rate was calibrated to represent a setting with observed (ie, detected) VL incidence of 5/10 000 capita (at equilibrium) before the start of improved detection, which for the baseline scenario translates to a true VL incidence of just over 10 cases/10 000/year and a mortality rate due to untreated VL of just over 5 cases/10 000/year. See Supplementary Appendix B for a formal description of the model equations, and Supplementary Appendix C for an overview of all biological parameter values and relevant references.

We developed 2 model variants: a deterministic variant defined in terms of a system of ordinary differential equations representing an infinitely large population, and a stochastic variant describing a discrete, finite set of individuals for whom transitions between disease stages are chance events based on the same transition rates as in the deterministic model variant. Both variants assume a closed, fixed-size population and were implemented in *pomp* (version 2.2.2.0) [28] using R (version 3.6.0) and RStudio (version 1.2.1335) software. The model code can be accessed through a public online repository at https://gitlab.com/erasmusmc-public-health/vl-detection-effort-model.

### Simulation Scenarios

First, we performed simulations with the deterministic model variant for various scenarios of improved detection, using a grid of values for population coverage of the improved detection strategy (0–100%, 1% increments) and reduction in detection delay among cases covered by the improved detection strategy (0–98%, 1% increments, relative to the baseline detection delay of 92 days). For each improved detection scenario, we predicted the true and observed VL incidence, mortality due to untreated (ie, undetected) VL, and the average duration of symptoms in detected cases after 5 years of improved case detection.

Second, to predict the impact of a potential relaxation of detection effort, we performed 10 000 stochastic simulations for a population size of 35 000 people (ie, the smallest block-level population size seen in the Indian subcontinent). Each stochastic simulation was initiated using a multinomial sample of 35 000 individuals with an expected state distribution as predicted for an equilibrium situation by the deterministic model variant before start of improved detection. Stochastic simulations were run with improved detection implemented at 80% population coverage with an achieved detection delay of 37 days (ie, a 60% reduction). A relaxation in detection effort was defined as a lowering of population coverage from 80% to 20%, while maintaining the achieved 60% reduction in detection delay, assuming that relaxation of detection effort does not affect the quality of the remaining effort because tools are still available and the healthcare workers are still primed. Relaxation of detection effort was assumed to occur in 2 situations: (1) after reaching the target of <1/10 000 observed VL cases for 3 consecutive years; or (2) after 5 years of improved control if program impact was unsatisfactory. For the first situation, we used the simulations that achieved the target for 3 years consecutively within 10 years of improved detection; the remainder of simulations (ie, not reaching the target within 10 years) was used for the second situation. After relaxation of detection effort, simulations were run for a further 5 years to monitor the changes in VL incidence (observed and true) and mortality.

## RESULTS

**Figure 1.**
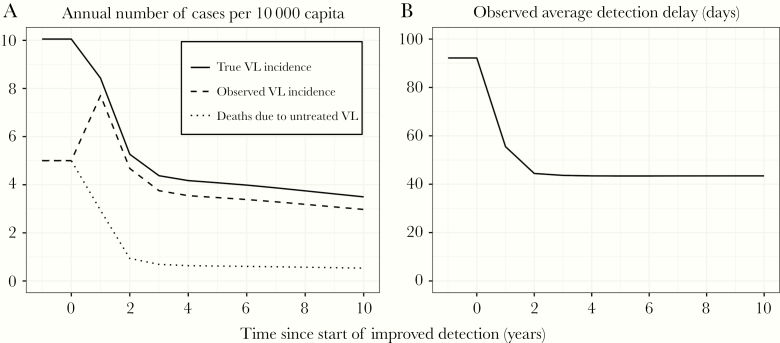
Deterministic model predictions for impact of improved case detection on visceral leishmaniasis (VL) incidence and mortality over time. Predictions reflect a setting where, before the start of improved detection, the annual observed incidence of VL was 5/10 000 capita, and half of all cases died before detection. Improved detection is assumed to result in a reduction of detection delay down to 37 days (60% reduction from 92 days) in 80% of the population covered by the improved detection program.

Figure 1 illustrates the impact of improved case detection on VL incidence and mortality over the course of 10 years, assuming 80% population coverage. The true VL incidence and mortality due to untreated VL were predicted to decline sharply within the first 3 years (Figure 1*A*), reflecting the impact of improved case detection on transmission. Observed VL incidence sharply increased during the first year of improved detection, approaching the true VL incidence, and then rapidly declined in the second and third year, followed by a stage of slow further decline. The predicted average duration of symptoms in detected cases (Figure 1*B*) declined immediately with the start of improved detection and stabilized after 2 years.

**Figure 2.**
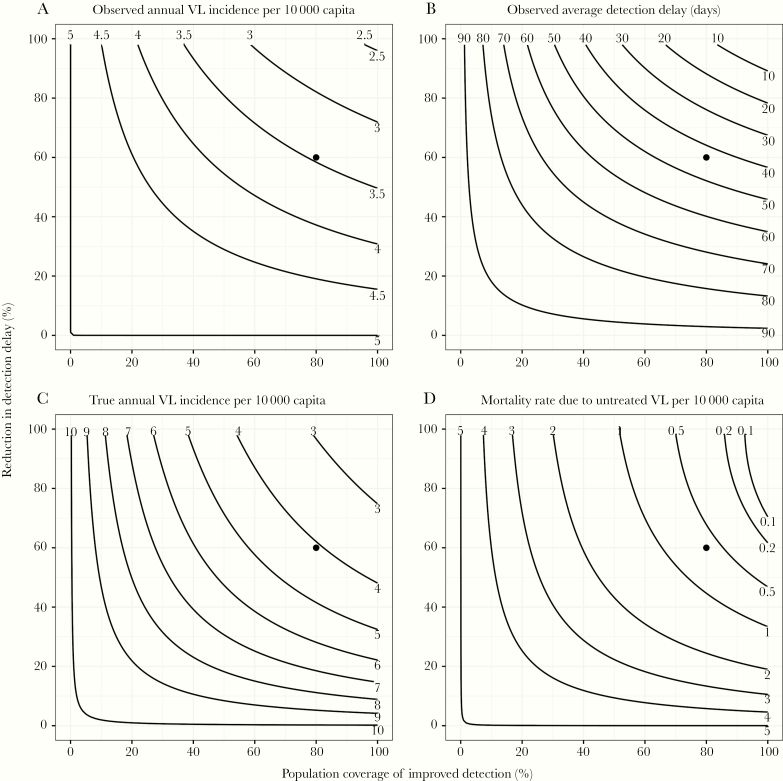
Contour plot of the model-predicted impact of 5 years of improved case detection at various levels of effectiveness on visceral leishmaniasis (VL). Model simulations represent a setting where, before the start of improved detection, the annual observed incidence of VL was 5/10 000 capita, and half of all cases died before detection. Improved detection is defined in terms of the proportion of the population covered by the program (x-axis) and the reduction in detection delay in the part of the population covered by program (y-axis), relative to a reference delay of 92 days without improved detection. Contour lines represent combinations of program coverage and reductions in detection delay that result in the same outcome after 5 years of improved detection. Panels represent different outcome metrics that can be directly measured (*A* and *B*) or not directly measured (*C* and *D*). Outcome metrics are based on both the covered and noncovered parts of the population. The point at 80% population coverage and 60% reduction in detection delay represents the scenario depicted in Figure 1.

Figure 2 summarizes the epidemiological situation after 5 years of improved case detection for various levels of detection effectiveness, again starting from the same baseline situation. Settings with poorly performing detection programs are represented by a reduction in detection delay (y-axis) of 0% (ie, a 92-day detection delay as in the baseline scenario) and/or 0% population coverage of the improved detection program (x-axis). In contrast, the top right corner of each panel represents a hypothetical ideal situation of maximum detection effectiveness in which the achieved treatment delays are shortest and the population coverage is highest. The solid circle in each panel represents the scenario depicted in Figure 1. Various combinations of program coverage and reductions in detection delay result in similar observed VL incidence (Figure 2*A*), with both parameters contributing approximately equally to the impact of improved case detection. Duration of symptoms in detected cases (Figure 2*B*) was predicted to decrease markedly with increasing program performance. A program coverage and a reduction in detection delay of both ≥60% ensured an overall detection delay of ≤50 days (among cases originating from both parts of the population covered and noncovered by improved detection). The difference between true VL incidence (Figure 2*C*) and the observed VL incidence (Figure 2*A*) decreased with increasing program performance (ie, toward the top-right corner). Mortality due to untreated VL (Figure 2*D*) decreased strongly with increasing program performance. A program coverage and a reduction in detection delay of both ≥65% ensured a mortality rate of <1/10 000/year. When detection delays are short, then ensuring increased population coverage has relatively more impact on reduction in mortality as demonstrated by the nearly vertical contour lines.

The stochastic version of the model highlights the important impact of chance effects related to the achievement of the target. In 13% of 10 000 stochastic simulations, the incidence of observed VL fell below 1/10 000 for 3 consecutive years during the first 10 years of the improved detection program (Supplementary Appendix D, panel *A*). These simulations represent the left tail of the expected distribution of outcomes for which the mean is the incidence trend predicted by the deterministic model (Figure 1). In the remaining 87% of simulations, the decline of the average VL incidence slowed down after 3 years of improved detection (as in Figure 1).

**Figure 3.**
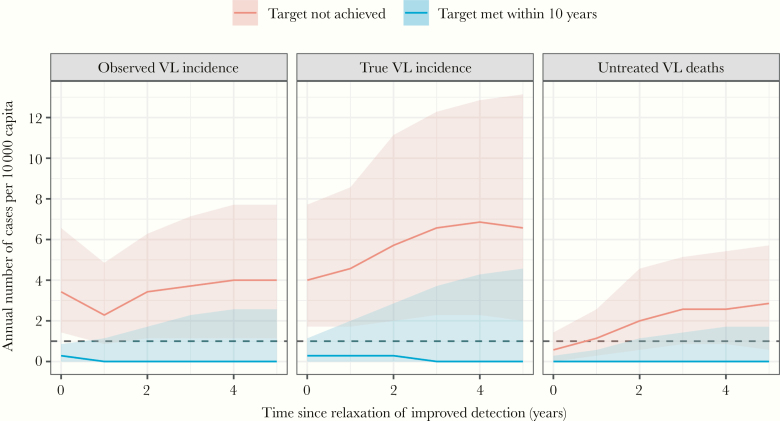
Stochastic model predictions for the number of visceral leishmaniasis (VL) cases and deaths when detection effort is relaxed after an initial period of improved detection. Simulations represent a setting where, before the start of improved detection, the annual observed incidence of VL was 5/10 000 capita, and half of all cases died before detection. Improved detection was defined as an average detection delay that is reduced from 92 to 37 days in 80% of the population covered by the improved detection program (as in Figure 1 and the point in Figure 2). Next, the detection effort was relaxed, either after reaching the target of <1/10 000 observed VL cases for 3 consecutive years (blue line and shaded band), or after 5 years if program impact was unsatisfactory (red line and shaded band). Relaxation of detection effort was defined as a decrease in program coverage from 80% to 20%. Lines and shaded bands represent the median and 80% confidence intervals of annual numbers from multiple stochastic simulations.

Figure 3 illustrates the potential impact of relaxing detection effort on VL incidence and mortality after an initial period of improved case detection. When detection was relaxed after meeting the target (ie, in 13% of 10 000 simulations; blue line and shaded band), transmission was either interrupted (55% of 13% of simulations with zero PKDL and VL cases), continued at levels with observed VL incidence <1/10 000 (18% of 13%), or resurged with observed VL incidence at or above 1/10 000 (27% of 13%) within the next 5 years. The predicted outcomes are shown in more detail in Supplementary Appendix D. In the subset of simulations with “unsatisfactory” impact of improved detection (ie, 87% of 10 000 simulations; red line and shaded band), a relaxation of detection effort resulted in an increase in true VL incidence and mortality. In contrast, the observed VL incidence declined during the first year after relaxation, after which it increases again. In 13% of the 87% of simulations, the observed VL incidence dropped to <1/10 000/year in the first year after relaxation of the detection effort (ie, the point where the lower bound of the red shaded band crosses the dashed horizontal line).

## Discussion

Our results demonstrate 5 key principles of VL control programs on the Indian subcontinent. First, successful implementation of improved case detection is expected to temporarily increase the observed VL incidence. However, finding and treating cases results in reduction of transmission so that the true case incidence and mortality fall. Second, successful case detection requires that reduction in detection delays covers the whole population. Third, there is an important role of chance in determining the likelihood of reaching and maintaining the control target. Fourth, when the control target is met, there is a high risk of resurgence of transmission if the detection effort is relaxed. Fifth, when little or no impact of improved detection is observed, a relaxation of the detection effort may result in a temporary reduction of observed VL incidence, sometimes even below the control target of 1/10 000/year, whereas the true VL incidence is actually increasing.

Clearly, observed VL incidence by itself is not a reliable indicator of program performance, because it is closely related to the detection effort, such that relaxation may even incorrectly suggest program improvement in the short run. Effective control has to be defined in terms of low case incidence combined with successful case detection and low average duration of symptoms. The presence of subpopulations who have longer detection delays (due to, eg, lower healthcare access and/or lower disease awareness) are important barriers to effective control. Our results show that the duration of symptoms in observed VL cases could serve as an additional indicator as it is temporally more directly related to the performance of case detection programs. The pattern in Figure 1B shows that the decrease quickly plateaus, which is not an indication that control is failing, but rather that detection effort is sustained. If the duration of symptoms in detected cases has not decreased significantly, then most likely the control target has only been seemingly (and temporarily) met because of poor case detection. Of course, the quality assurance accuracy of reported detection delays remains challenging, given the fact that individuals often attend multiple clinics before being diagnosed with VL.

An independent measure of case detection effort and success (ie, if a case is there, will it be diagnosed and how long will it take) would underpin the current interventions. It would also avoid potential perverse incentives (eg, lowering detection effort or reporting fewer cases to reach the control target). Currently, there is no systematic data collection on measures of diagnostic effort (eg, number of suspected cases tested or number of cases of splenomegaly tested). Requiring programmatic reporting of such data would keep VL in the clinic focus even when there are zero cases, and would also provide denominators to estimate the rate of VL detection. A small proportion of PKDL cases arise without previous treatment, so reporting these separately from PKDL cases with known VL history would provide a measure of the relative incidence of undiagnosed VL. Other approaches would require development of systems beyond the current program (eg, postmortem measurements), which are unlikely to be initiated solely for the VL program.

A successful detection program, in which most VL cases are diagnosed promptly, means that the observed VL incidence more accurately represents the true state of the population. In particular, if the VL incidence target is met due to reduction in transmission through diagnosis and treatment, then it is guaranteed that the true (unobserved) mortality due to VL is also low (Figure 2). A successful detection program involves many processes including community and clinical awareness, access to healthcare, and availability of diagnostics, and we have not included any of these details, but we show that it is important that reductions in detection delay have wide population coverage. This is relevant when considering active case detection or other activities targeted to “hotspots,” and to ensure that they do not result in sections of the population with reduced detection that can continue to support transmission.

It has been recognized that VL diagnoses are clustered in time and space, and pursuing active case detection in communities in which further cases are expected exploits this epidemiological observation. For instance, in India the control program focuses on finding febrile patients in the vicinity of index VL cases. Xeno-monitoring—that is, surveillance of vectors for presence of infection and infectiousness—is another avenue being actively considered. Given that there appears to be little transmission from asymptomatic cases, the presence of infected sandflies might be good evidence of a case of infectious VL or PKDL in the community. However, this needs to be confirmed.

Our deterministic model suggests that the observed VL incidence cannot reach <1/10 000 within 5 years of improved control (Figure 2), but stochastic model predictions suggest that the control targets can be met in a proportion of situations with similar or lower VL incidence than considered here (precontrol annual VL incidence of 5/10 000 capita). The simulations also show that even when targets are achieved there is a chance of resurgence. This difference highlights the deficiency of deterministic models to adequately capture stochastic effects in populations of finite size. Some of the parameters in the model have had to be inferred, so we focus our attention on the qualitative, rather than quantitative, results.

The achievement of the control target in various field settings with similar or even higher precontrol VL incidence than considered may be explained by concomitant changes in human exposure to sandfly bites, for example, due to successful use of indoor residual spraying and/or other factors that affect sandfly biology, which were not considered in the model here. Conclusions with regard to (relaxation) of detection effort do not depend on the above factors.

We have assumed that transmission within the population is homogeneous, that is, that each individual is equally likely to transmit to each other individual. This is a simplification of reality, and given the role of relatively short-range vectors, the transmission dynamics of VL are likely better captured by considering meta-populations (eg, populations of people within separate villages), and we are actively pursuing this hypothesis. How the processes we have studied here interact with transmission at multiple scales is not immediately clear, but we are confident that our underlying results are robust.

It is becoming clear that only VL and PKDL cases can transmit significantly to sandflies, but there remain many important parameter values, such as proportion developing different types of PKDL (nodular, popular, etc), their infectiousness, and their duration, for which good data are still accruing [3]. Similarly, the potential roles of longer-term immunity following VL and asymptomatic infection are largely unknown. However, these will largely influence longer-term dynamics, and the shorter-term patterns that we explore here are dominated by 1 infection per host and do not include the recycling of hosts through the susceptible class.

In conclusion, we show that VL incidence on its own is not a reliable indicator of the performance of case detection programs. Unless transmission is truly interrupted, relaxation of detection effort will result in a temporary reduction of observed VL incidence while true VL incidence and mortality rise immediately. Therefore, continued case detection is pivotal for sustained control of VL. Our findings indicate that the average duration of symptoms in detected cases is a useful indicator of the performance of case detection programs, although there is also a need for independent measures of case detection effort, such as number of suspected cases screened for VL, to avoid perverse incentives.

## Supplementary Data

Supplementary materials are available at *The Journal of Infectious Diseases* online. Consisting of data provided by the authors to benefit the reader, the posted materials are not copyedited and are the sole responsibility of the authors, so questions or comments should be addressed to the corresponding author.

jiz644_suppl_Supplementary-Appendix_A

jiz644_suppl_Supplementary-Appendix_B

jiz644_suppl_Supplementary-Appendix_C

jiz644_suppl_Supplementary-Appendix_D

## Notes

***Author contributions.*** Conception and design: L. E. C., G. F. M., S. J. d. V., J. M. P. Programming: L. E. C., J. M. Analysis: L. E. C., J. M. A. Interpretation: all authors. Drafting of manuscript: L. E. C., E. A. L. R., E. R. A., J. M. P. Critical review and revision of manuscript: all authors.

***Disclaimer.*** The funders had no role in study design, data collection and analysis, decision to publish, or preparation of the manuscript. The views, opinions, assumptions, or any other information set out in this article are solely those of the authors and should not be attributed to the funders or any person connected with the funders.

***Financial support.*** All authors gratefully acknowledge funding of the NTD Modelling Consortium by the Bill & Melinda Gates Foundation (grant number OPP1184344). L. E. C. further acknowledges funding from the Dutch Research Council (grant number 016.Veni.178.023).

***Supplement sponsorship.*** The authors acknowledge funding of the NTD Modelling Consortium by the Bill and Melinda Gates Foundation (OPP1184344).

***Potential conflicts of interest.*** All authors: No reported conflicts of interest.
