# Supplementary material for: Impact of Changes in Detection Effort on Control of Visceral Leishmaniasis in the Indian Subcontinent
Source: J Infect Dis. 2019 Dec 16;221(Suppl 5):S546–53. doi: 10.1093/infdis/jiz644 (PMC7289545; doi:10.1093/infdis/jiz644)

Appendix A. Schematic representation of the mathematical model for transmission and improved detection of visceral leishmaniasis (VL). The model is a simplified version of earlier transmission models [16–18], keeping only the processes in the model that are relevant to the impact of improved detection of VL cases. Population coverage of improved detection is represented by fraction f_d. Each compartment for the symptomatic stage ($I_{1,1-3}$ and $I_{2,1-3}$) is divided in three equal parts for progress towards death due to untreated VL, assuming that time till death due to untreated VL follows an Erlang distribution with shape 3. Symptomatic cases can be detected at any stage during progress towards death. The hazard of dying from untreated VL before detection is represented by rate μ_{VL}, which is the same for all individuals, regardless of whether they are covered by improved or baseline detection. However, because detection rate ρ_{I1} in the population covered by improved detection is higher than detection rate ρ_{I2} in the population covered by baseline detection, the effective risk of dying from untreated VL is higher in the latter.

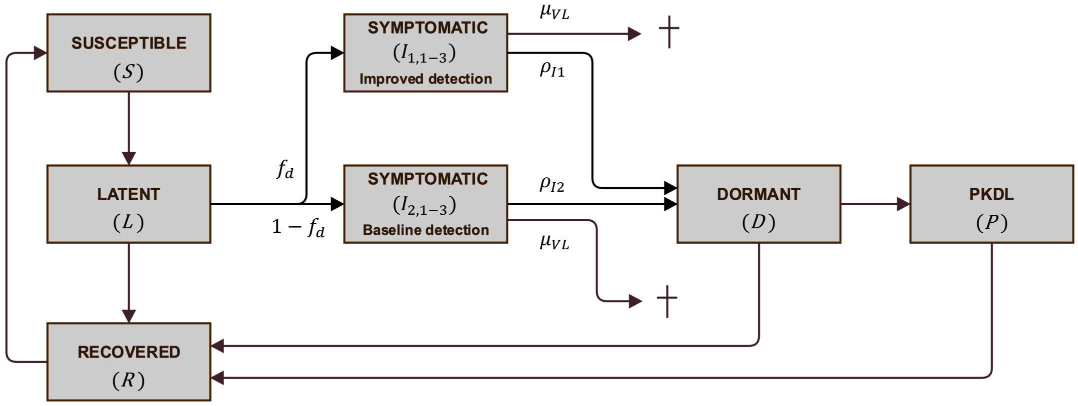

Supplement: jiz644_suppl_Supplementary-Appendix_A [file jiz644_suppl_supplementary-appendix_a.pdf]
