# Supplementary material for: Impact of Changes in Detection Effort on Control of Visceral Leishmaniasis in the Indian Subcontinent
Source: J Infect Dis. 2019 Dec 16;221(Suppl 5):S546–53. doi: 10.1093/infdis/jiz644 (PMC7289545; doi:10.1093/infdis/jiz644)
Supplement: jiz644_suppl_Supplementary-Appendix_B [file jiz644_suppl_supplementary-appendix_b.pdf]

Appendix B. Model equations

Below follows the set of equations that describe both the deterministic and stochastic model variants. For an overview and explanation of the symbols used in the equations, see the table on the next page.

$$\frac{dS}{dt} = \mu \cdot N + 3 \cdot \mu_{VL} \cdot \left(I_{1,3} + I_{2,3}\right) + \rho_R \cdot R - (\mu + \lambda) \cdot S$$

$$\frac{dL}{dt} = \lambda \cdot S - (\rho_L + \mu) \cdot L$$

$$\frac{dI_{1,1}}{dt} = f_d \cdot f_s \cdot \rho_L \cdot L - (\rho_{I1} + \mu + 3 \cdot \mu_{VL}) \cdot I_{1,1}$$
$$\frac{dI_{1,2}}{dt} = 3 \cdot \mu_{VL} \cdot I_{1,1} - (\rho_{I1} + \mu + 3 \cdot \mu_{VL}) \cdot I_{1,2}$$
$$\frac{dI_{1,3}}{dt} = 3 \cdot \mu_{VL} \cdot I_{1,2} - (\rho_{I1} + \mu + 3 \cdot \mu_{VL}) \cdot I_{1,3}$$

$$\frac{dI_{2,1}}{dt} = (1 - f_d) \cdot f_s \cdot \rho_L \cdot L - (\rho_{I2} + \mu + 3 \cdot \mu_{VL}) \cdot I_{2,1}$$
$$\frac{dI_{2,2}}{dt} = 3 \cdot \mu_{VL} \cdot I_{2,1} - (\rho_{I2} + \mu + 3 \cdot \mu_{VL}) \cdot I_{2,2}$$
$$\frac{dI_{2,3}}{dt} = 3 \cdot \mu_{VL} \cdot I_{2,2} - (\rho_{I2} + \mu + 3 \cdot \mu_{VL}) \cdot I_{2,3}$$

$$\frac{dD}{dt} = \sum_{g=1}^{2} \sum_{m=1}^{3} \rho_{Ig} \cdot I_{g,m} - (\rho_D + \mu) \cdot D$$

$$\frac{dP}{dt} = f_P \cdot \rho_D \cdot D - (\rho_P + \mu) \cdot P$$

$$\frac{dR}{dt} = (1 - f_s) \cdot \rho_L \cdot L + (1 - f_P) \cdot \rho_D \cdot D + \rho_P \cdot P - (\rho_R + \mu) \cdot R$$

$$\lambda = \beta \left(\beta_p P + \sum_{g=1}^{2} \sum_{m=1}^{3} I_{g,m} \right) \Big/ N$$

$$N = S + L + D + P + R + \sum_{g=1}^{2} \sum_{m=1}^{3} I_{g,m}$$

Symbol	Description
S	Susceptible
L	Latent infection
$I_{g,m}$	Symptomatic infection (visceral leishmaniasis), with $g \in \{1,2\}$ indicating the group membership with regard to whether or not the individual is covered by the improved detection programme (1 = yes, 2 = no), and $m \in \{1,2,3\}$ indicating the mth compartment of the Erlang distribution for progress until death due to untreated disease.
D	Dormant
P	Post-kala-azar dermal leishmaniasis
R	Recovered
N	Total human population size
μ	Background mortality rate
μ_{VL}	Excess mortality rate due to untreated visceral leishmaniasis, assuming that time until death follows an Erlang distribution with shape 3 (i.e. the $m \in \{1,2,3\}$ compartments in $I_{g,m}$).
λ	Force of infection
β	Overall transmission rate, incorporating sandfly density, sandfly biting rate, and transmission probability from fly to human
β_P	Infectiousness of post-kala-azar dermal leishmaniasis relative to visceral leishmaniasis
ρ_L	1 / Average duration of latent infection
ρ_{Ig}	1 / Average duration until detection and treatment of visceral leishmaniasis in group $g \in \{1,2\}$
ρ_D	1 / Average duration of the dormant stage, such that if a case develops post-kala-azar dermal leishmaniasis $f_P \cdot \rho_D$ is 1 / the average duration between treatment of visceral leishmaniasis and onset of post-kala-azar dermal leishmaniasis, and if no post-kala-azar dermal leishmaniasis is developed $(1 - f_P) \cdot \rho_D$ is 1 / the average duration until full recovery (immunity) after treatment of visceral leishmaniasis.
ρ_P	1 / Average duration of post-kala-azar dermal leishmaniasis
ρ_R	1 / Average duration of the recovered (immune) stage
μ	Human background mortality rate
μ_{VL}	Excess mortality due to visceral leishmaniasis
f_d	Proportion of humans in whom symptomatic infection is more easily detected
f_s	Proportion of infections that progress to visceral leishmaniasis
f_P	Proportion of visceral leishmaniasis cases that develop post-kala-azar dermal leishmaniasis
