# Supplementary material for: Impact of Changes in Detection Effort on Control of Visceral Leishmaniasis in the Indian Subcontinent
Source: J Infect Dis. 2019 Dec 16;221(Suppl 5):S546–53. doi: 10.1093/infdis/jiz644 (PMC7289545; doi:10.1093/infdis/jiz644)
Supplement: jiz644_suppl_Supplementary-Appendix_C [file jiz644_suppl_supplementary-appendix_c.pdf]

Appendix C. Parameter values used in simulations

Parameter	Symbol	Value	Source
Average duration of latent infection (days)	$1/\rho_L$	150	[18]
Average duration dormant stage (months)	$1/\rho_D$	21	[24–26]
Average duration PKDL (years)	$1/\rho_P$	5	[24]
Average duration recovered stage (years)	$1/\rho_R$	5	[18]
Transmission rate	β	92.2	Calibrated to produce an observed annual VL incidence of 5 per 10,000 capita in equilibrium
Relative infectivity of VL	-	1	(reference value)
Relative infectivity of PKDL	β_P	0.9	[3]
Percentage of latently infected that progress to VL (%)	f_s	3	[19]
Percentage of dormant infections that progress to PKDL (%)	f_P	5	[2]
Excess mortality rate in untreated VL cases (1/day)	μ_{VL}	1/189	Jointly calibrated with the baseline detection rate (such that the average time until death is 150 days and 50% of VL cases die undetected, conditional on the assumption that time until death due to untreated VL follows an Erlang distribution (k=3).
Baseline detection rate for VL with unimproved detection (1/day)	ρ_{Ig}	1/243	Jointly calibrated with the excess mortality rate such that the average time until death is 150 days and 50% of VL cases die undetected, conditional on the assumption that time until death due to untreated VL follows an Erlang distribution (k=3).
Coverage of improved detection strategy (%)	f_d	0-100	Assumption
Reduction in detection delay in sub-population covered by improved detection strategy (%)	Function of multiple parameters[a]	0-98	Assumption
Background mortality rate (1/year)	μ	1/68	Based on average lifespan at birth in rural Bihar, 2010–2014 [27].

[a] A function of detection rates ρ_{I1} and ρ_{I2}, background mortality rate μ, and excess mortality rate μ_{VL}: $\text{delay}_1/\text{delay}_2$, where $\text{delay}_g = \left(\frac{\rho_{Ig}}{\rho_{Ig}+\mu+M\cdot\mu_{VL}}\right)\sum_{m=1}^{M}\left[\left(\frac{M\cdot\mu_{VL}}{\rho_{Ig}+\mu+M\cdot\mu_{VL}}\right)^{m-1}\left(\frac{m}{\rho_{Ig}+\mu+M\cdot\mu_{VL}}\right)\right]$, with $M = 3$ (i.e. the number of chained compartments in $I_{g,m}$ for progression towards death due to untreated VL). The first term represents the probability of a case being detected while in any of the compartments $I_{g,m}$. The second term represents the probability that VL cases remain undetected and survive up to the mth compartment of $I_{g,m}$, times the average duration of symptoms of individuals that are detected while in that compartment.
