# Supplementary material for: Impact of Changes in Detection Effort on Control of Visceral Leishmaniasis in the Indian Subcontinent
Source: J Infect Dis. 2019 Dec 16;221(Suppl 5):S546–53. doi: 10.1093/infdis/jiz644 (PMC7289545; doi:10.1093/infdis/jiz644)

Appendix D. Stochastic model predictions for observed annual incidence of visceral leishmaniasis (VL) when detection effort is relaxed after an initial period of improved detection. Simulations represent a setting where, before the start of improved detection, the annual observed incidence of VL was 5 per 10,000 capita, and half of all cases died before detection. Improved detection was defined as an average detection delay that is reduced from 92 to 37 days in 80% of the population covered by the improved detection programme (as in Figure 1 and the point in Figure 2). Panel A shows model predictions for a situation with continued improved detection, stratified by the year when the target of <1/10,000 observed VL cases was met for three consecutive years (coloured lines). Panel B show model predictions for a scenario where the detection effort was relaxed after reaching the target for three consecutive years or after five years if programme impact was unsatisfactory (i.e. the pink representing simulations that did not meet the target within ten years). Relaxation of detection effort was defined as lowering programme coverage from 80% to 20%. Lines represent the mean of repeated stochastic simulations. Percentages in panel A indicate the proportion of stochastic simulations in each stratum.

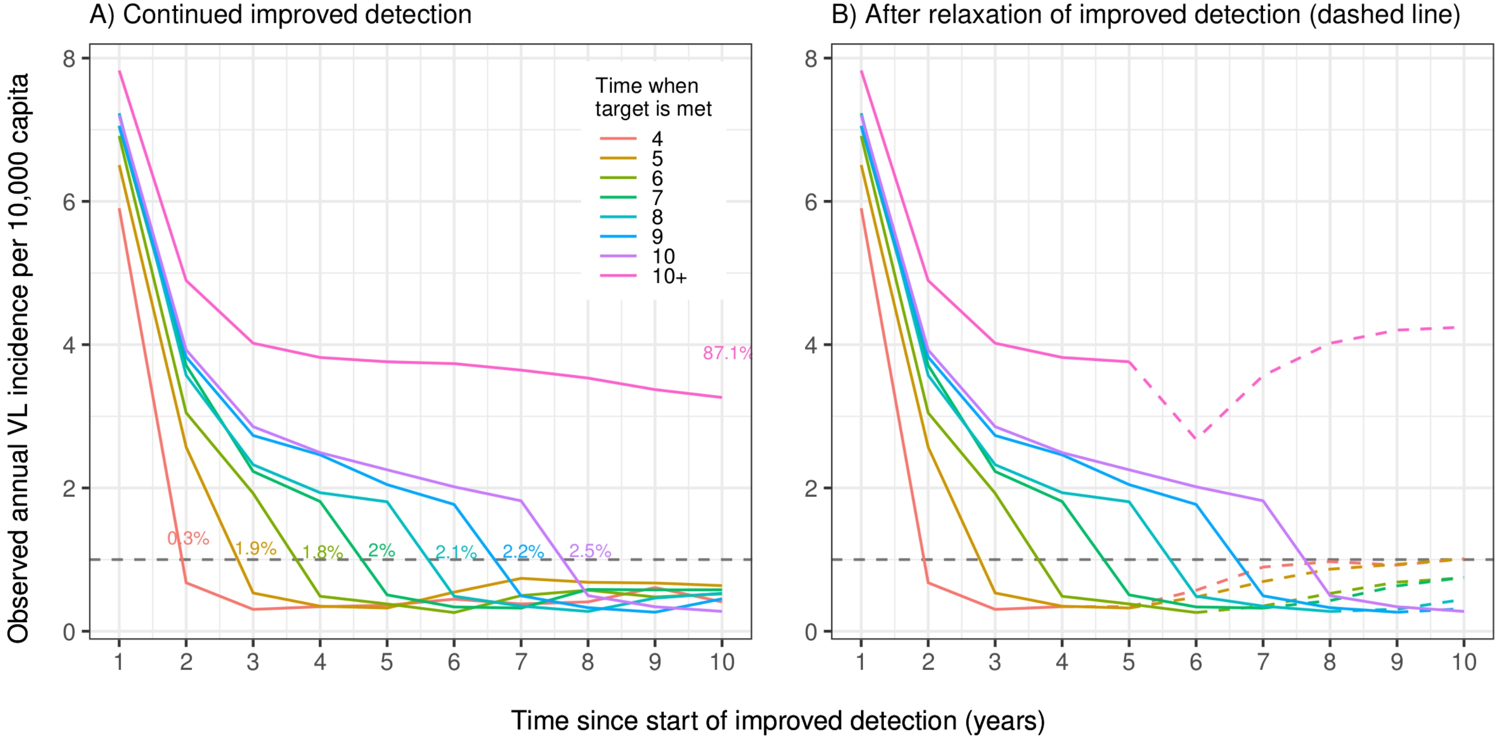

Supplement: jiz644_suppl_Supplementary-Appendix_D [file jiz644_suppl_supplementary-appendix_d.pdf]
